# High diversity droplet microfluidic libraries generated with a commercial liquid spotter

**DOI:** 10.1038/s41598-021-83865-y

**Published:** 2021-02-23

**Authors:** Jesse Q. Zhang, Christian A. Siltanen, Ata Dolatmoradi, Chen Sun, Kai-Chun Chang, Russell H. Cole, Zev J. Gartner, Adam R. Abate

**Affiliations:** 1 Department of Bioengineering and Therapeutic Sciences, University of California San Francisco, San Francisco, CA USA; 2 UC Berkeley-UCSF Graduate Program in Bioengineering, University of California San Francisco, San Francisco, CA USA; 3 Scribe Biosciences, Inc., San Francisco, CA USA; 4 Department of Pharmaceutical Chemistry, University of California San Francisco, San Francisco, CA USA; 5 Chan Zuckerberg Biohub, San Francisco, CA USA; 6 California Institute for Quantitative Biosciences, University of California San Francisco, San Francisco, CA USA

**Keywords:** Biomedical engineering, Microfluidics

## Abstract

Droplet libraries consisting of many reagents encapsulated in separate droplets are necessary for applications of microfluidics, including combinatorial chemical synthesis, DNA-encoded libraries, and massively multiplexed PCR. However, existing approaches for generating them are laborious and impractical. Here, we describe an automated approach using a commercial array spotter. The approach can controllably emulsify hundreds of different reagents in a fraction of the time of manual operation of a microfluidic device, and without any user intervention. We demonstrate that the droplets produced by the spotter are similarly uniform to those produced by microfluidics and automate the generation of a ~ 2 mL emulsion containing 192 different reagents in ~ 4 h. The ease with which it can generate high diversity droplet libraries should make combinatorial applications more feasible in droplet microfluidics. Moreover, the instrument serves as an automated droplet generator, allowing execution of droplet reactions without microfluidic expertise.

## Introduction

Droplet microfluidics uses micron-scale water-in-oil emulsions to conduct assays at high throughput and using minimal reagents. These qualities allow applications that would be difficult or impossible with conventional pipetting-based liquid handling. The most successful implementations of droplet microfluidics are high throughput bioassays that analyze large numbers of discrete components, such as DNA molecules^1^, functionalized beads^2,3^, or cells^4,5^. For example, in enzyme screening, the sample can consist of microbes expressing variants from a library^6^, while in single cell genomics, it can consist of cells with distinctive molecular features^7^. In applications like these, droplets containing distinct discrete components but uniform reagent conditions allow high throughput quantitative analysis. For example, a uniform catalytic measurement can score enzyme variants by activity^8^, while uniform reaction conditions can barcode and sequence thousands of distinct single cell transcriptomes^9^. However, other applications require the reagents within the droplets to also vary. For example, combinatorial chemistry synthesizes chemical libraries by mixing a fixed set of input reactants in different combinations^10^. Similarly, drug screens test hundreds of compounds, individually or in combination, against model systems to elicit a desired response^11^. Such combinatorial applications would greatly benefit from the speed and efficiency of droplet microfluidics, but have been limited by the difficulty of rapidly generating libraries of distinct reagent droplets.

A common strategy for generating droplet libraries is to emulsify each solution sequentially and pool the products^11,12^. When performed manually, however, this approach is only practical for small numbers of reagents. Automated emulsification is more scalable but requires custom robotics that interface with the microfluidic device, which can be technically challenging^13,14^. Both approaches are limited by the speed of one drop maker, and contamination is a major issue as all solutions are emulsified by the same device. Parallel devices avoid contamination by emulsifying each solution in its own drop maker and are much faster since hundreds of devices can operate in unison^15,16^. However, parallel devices are difficult to design, build, and operate, and prone to failure due to imperfect fabrication or dust clogging some drop makers. The resultant emulsions often lack key reagents from the library and contain large, polydispersed droplets that can make them unsuitable for further use. Thus, generating libraries of monodispersed droplets from hundreds of different reagents remains the major barrier to applying droplet microfluidics to combinatorial applications.

In this paper, we describe simple and fast generation of high diversity droplet libraries with a commercial reagent spotter. Our approach uses the Scienion SciFlexArrayer, an instrument designed to print droplets onto solid substrates^17,18^. Instead, we use it to dispense droplets into a surfactant-laden oil bath, thereby generating stable, monodispersed droplets. Because the instrument is designed for commercial use, it has attractive features for library generation, including automated interfacing with well plates and rigorous washing of dispensing surfaces between samples. The piezo-electric droplet generator is controllable, allowing droplet volumes from 50 to 800 pL to be generated. It is fast, generating monodisperse droplets up to 500 Hz, thus taking only a few hours to create millions of droplets of hundreds of reagents. The droplet generation is on demand, allowing the exact proportion of each reagent in the final library to be defined. The instrument can also perform assays normally requiring a microfluidic device and the associated expertise. As a demonstration, we use it to perform digital PCR, though other assays for enzyme screening, single cell analysis, and analyte quantitation are feasible. The speed, reliability, and ease of this approach for generating high diversity droplet libraries should make combinatorial applications more accessible.

## Results and discussion

**Figure 1:**
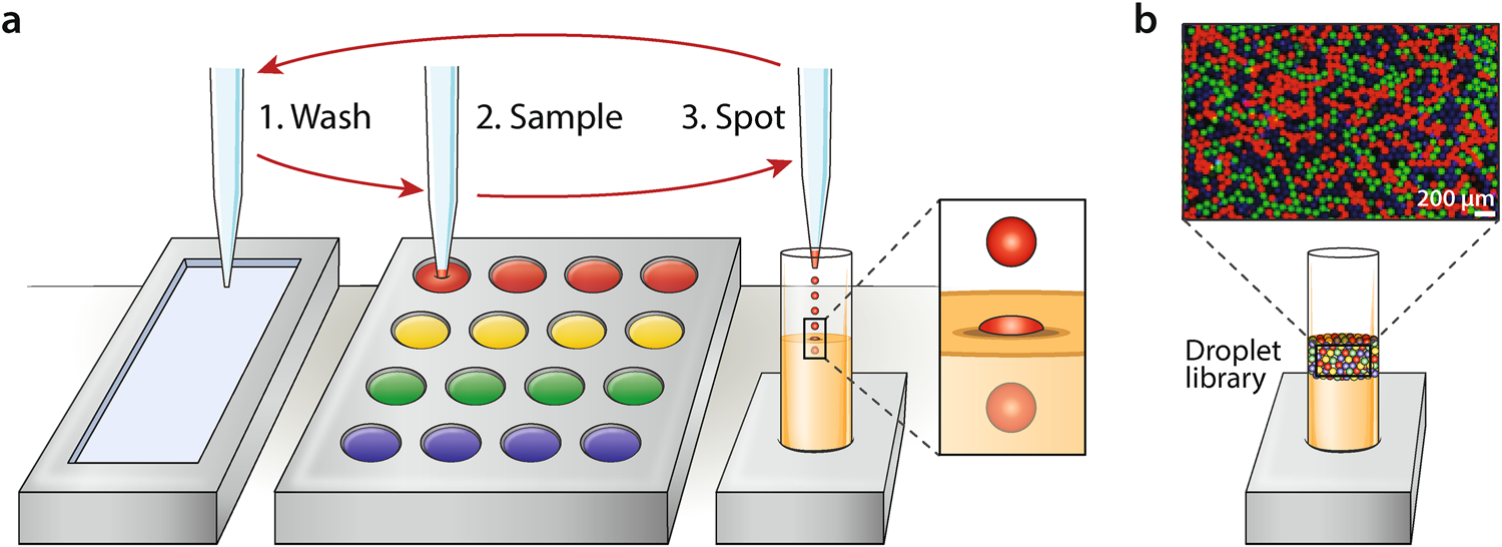
Automated liquid handling and dispensing with commercial drop in air printer for generation of monodisperse droplet libraries. (**a**) Each sample is emulsified by operating the printer’s capillary nozzle in a three-step cycle. 1. The nozzle moves to the wash tray and the previous contents in the nozzle are ejected; 2. The nozzle moves to the sample plate and suctions several microliters of sample; 3. The nozzle moves to the oil bath and ejects droplets into an oil bath, after which it moves to the wash tray. (**b**) Droplet libraries generated in this fashion are monodisperse and analogous to those generated by a microfluidic device.

Commercial piezo-electric droplet printing, which normally prints onto solid substrates, can be instead used to make controlled emulsions by dispensing into an oil bath. Automated emulsification is accomplished using a three-step cycle repeated for each reagent (Fig. 1a). The capillary nozzle of the printer moves to the wash tray, where residual sample from previous cycles is removed; this is an essential step to minimize cross contamination. The nozzle moves to the sample plate, typically a 96 or 384 well plate containing the different solutions to be emulsified, loading the desired amount of reagent into the capillary. These standard well plates are often used to store compound libraries. Finally, the nozzle moves above the oil bath, where it ejects droplets of reagent. The droplets pierce the oil layer and are coated by surfactant, pooling beneath the oil-air interface (Fig. 1b). The droplets are produced at 50–900 µL/h depending on size and instrument settings, such that a library totaling ~ 5 mL can be created in ~ 5.5 h. Switching between samples adds instrument movement, capillary wash, and sampling time of about 3 min per reagent. For example, a 1 mL library of 3.3 million 300 pL droplets comprising 10 reagents would take ~ 2.5 h to produce, while the same volume of 100 reagents would take ~ 7 h.

**Figure 2:**
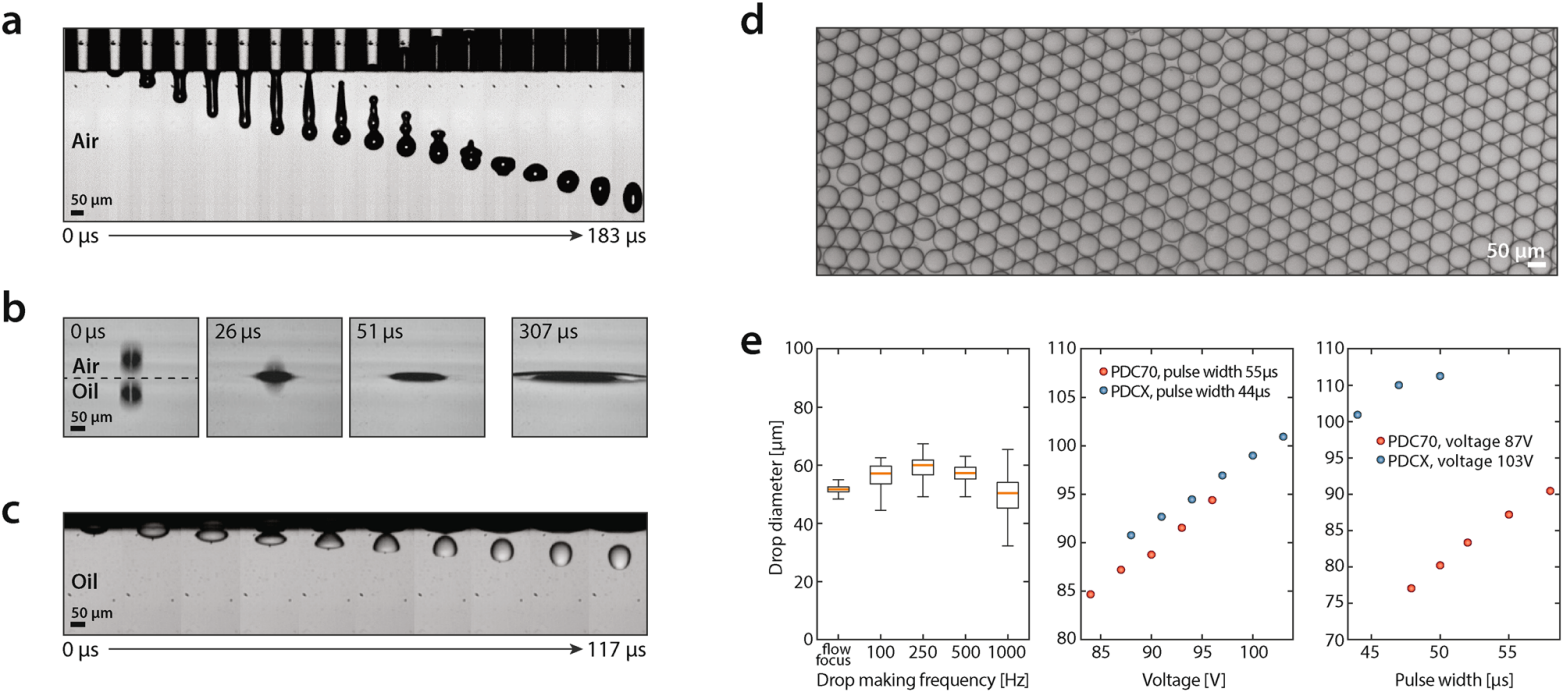
Drop-in-air printing into oil is rapid, reliable, and tunable. (**a**) Time-lapse imagery of the droplet being ejected from the capillary tube into air by acoustic waves. (**b**) Impact of droplet into the oil layer. (**c**) Behavior of the droplet once it pierces the oil layer. (**d**) Micrograph of droplets generated at 300 Hz. (**e**) Droplet size distribution as a function of acoustic wave frequency (left), voltage (center), and pulse width (right).

The SciFlexArrayer generates droplets on demand from static fluids maintained in the dispensing capillary via actuation of piezo-electric driven pressure pulses (Fig. 2a). Initially, the capillary is filled to the tip with the dispensing liquid; when the pulse is applied, a droplet bulges from the tip and detaches. The remaining liquid retracts up the capillary before refilling and coming to rest at the tip, where the cycle can repeat. The ejected droplet continues forward due to its inertia, piercing the top of the oil and generating ripples traveling outward from the point of impact (Fig. 2b). Once in the oil, the droplet assumes a deformed shape due to viscous drag until its inertia is fully damped and it comes to rest, at which point it floats up due to its buoyancy (Fig. 2c).

When operating over an exactly repeating duty cycle, this mechanism generates droplets of identical size that are comparable to ones produced by a microfluidic device (Fig. 2d). Surfactants stabilize the droplets; if omitted, droplets can coalesce in the oil (Movie S1). We find that 2% w/v fluorosurfactant prevents coalescence, even if droplets collide soon after entering the oil (Movie S2). Depending on droplet generation speed or water and oil composition, the surfactant may not stabilize the droplets before collision, which could lead to coalescence. Under such circumstances, surfactant, droplet, and oil composition must be optimized to minimize coalescence. One approach is to lower the droplet generation rate to prevent sequential droplets from contacting before they are stable. For example, we find that for water droplets generated at 500 Hz, monodispersity is high, but coalescence occurs at higher frequencies, resulting in polydispersity (Fig. 2e, left). We also observe that agitating the oil bath during printing by adding a stir bar reduces coalescence by reducing the chance that sequential droplets impact (Fig. S1) and that droplet printing is robust to the distance between the nozzle and oil bath (Fig. S2). Lastly, we demonstrate minimal carry-over between samples by including a wash step (Fig. S3).

Since the minimum volume of an ejected droplet scales with capillary diameter, smaller capillaries generate smaller droplets^19^. For a given capillary size, droplet diameter can be varied by tuning pulse amplitude and width. Increasing amplitude at fixed pulse width yields a linear increase in droplet diameter (Fig. 2e, center). Increasing pulse width at fixed amplitude also yields a linear increase in droplet diameter (Fig. 2e, right). In addition, droplet volume remains stable over the modulated parameters, with a measured coefficient of variation between 0.3% and 1.7%. We modulate drop volumes between 76 and 94 µm and 91 and 106 µm for the two different capillaries. Thus, tuning both parameters and using different sized capillaries affords a wide range of controlled droplet diameters. This demonstrates that the SciFlexArrayer is an effective instrument for generating monodispersed emulsions of controlled size without microfluidics. Thus, it should be useful to labs interested in conducting monodispersed droplet reactions but lacking microfluidic expertise.

**Figure 3:**
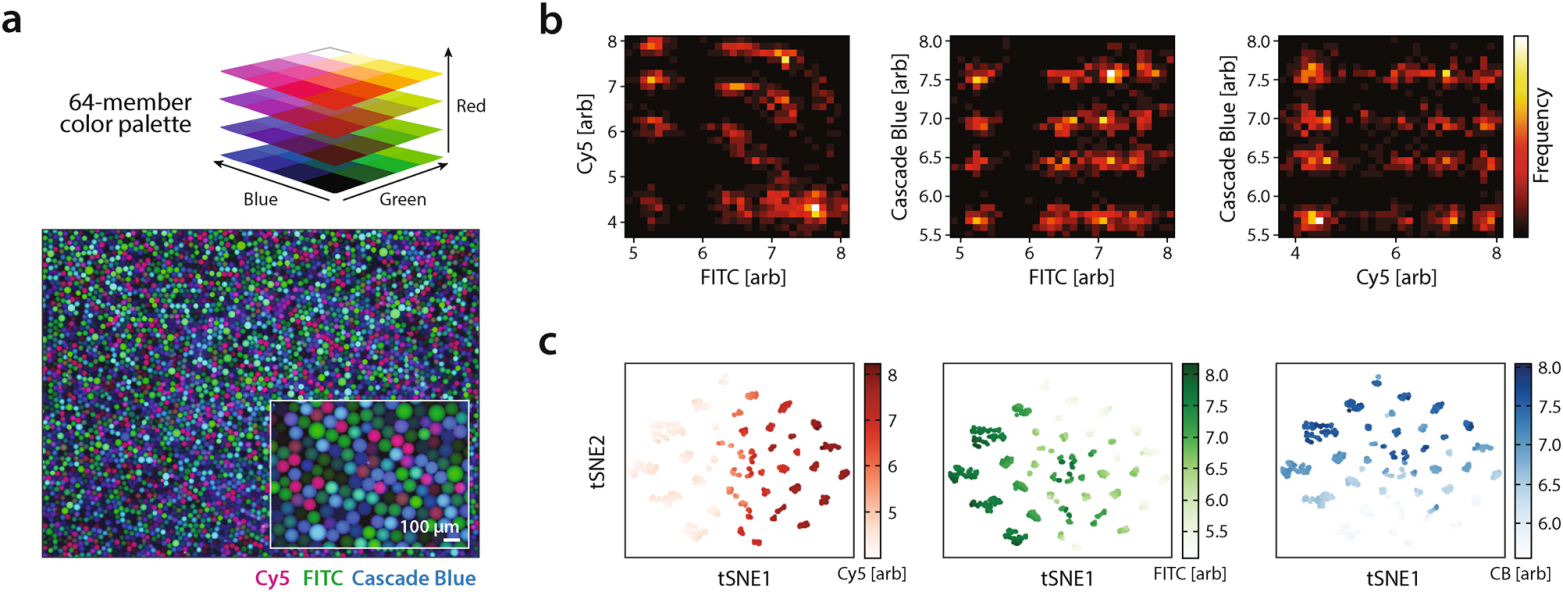
Emulsification of a large optically encoded library. (**a**) A 64-member color palette of all possible combinations of 4 levels of blue, green, and red dyes is emulsified with the printer. (**b**) Based on images of the emulsion, brightness in the Cascade Blue (CB), FITC, and Cy5 channels is extracted from each droplet and visualized as a series of 2-D heatmaps. (**c**) To identify distinct droplet populations, drops are filtered and analyzed by T-distributed stochastic neighbor embedding (tSNE). The raw fluorescence data is overlaid on each analyzed droplet on the tSNE plot.

In addition to generating controlled emulsions, a major value of the SciFlexArrayer is its ability to emulsify solutions stored in well plates with full automation. To demonstrate this, we construct a reagent set comprising 3 dyes at 4 concentrations, yielding 64 combinations (Fig. 3a, top). We image the constructed emulsion in 3 fluorescence channels (Fig. 3a, bottom). We extract intensity values from the center of each droplet and generate heatmaps (Fig. 3b). We generate histograms for each of the fluorescence channels (Fig. S4a) observing peaked distributions for all 4 dye concentrations. Due to crosstalk between FITC and Cy5, the distributions overlap for some droplets. To facilitate visualization of this 3D data, we perform a dimensionality reduction using T-distributed stochastic neighbor embedding (tSNE)^20^, filtering out droplets that do not cluster at the expected dye concentrations (Fig. S4b), obtaining 54 clusters (Fig. 3c). The unresolved clusters result from low intensity droplets and cross talk between dyes, which become difficult to resolve by widefield fluorescence microscopy due to light variation at the periphery of the images (Fig. 3a). Overall, this library of ~ 2 mL total volume took ~ 4 h to generate from the well plate, demonstrating the effectiveness of this approach for transforming well plate libraries into droplet libraries suitable for microfluidic use.

**Figure 4:**
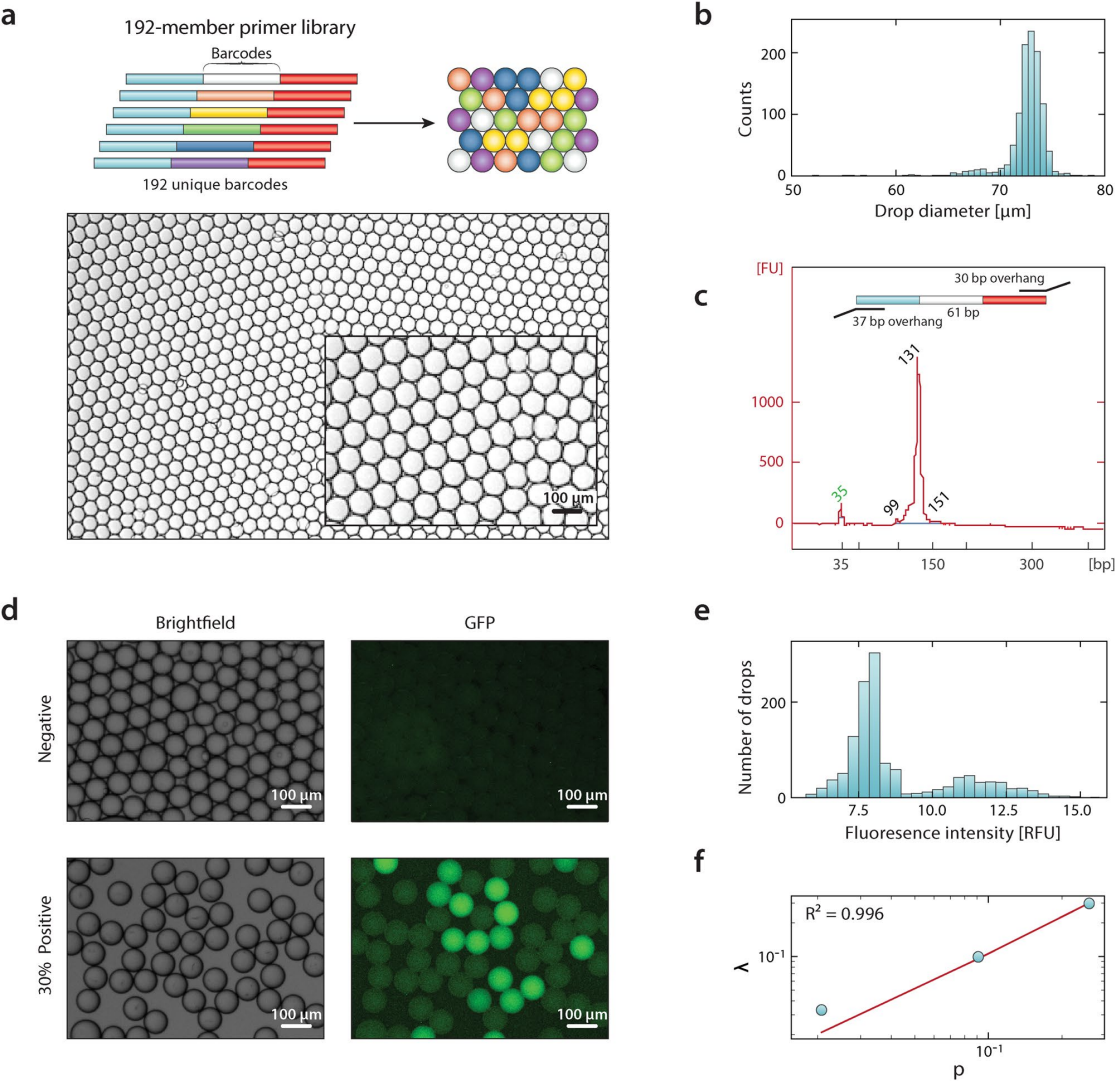
Amplification of oligonucleotides encapsulated within droplet libraries. (**a**) A library consisting of oligos consisting of one of 192 barcode sequences flanked by constant regions is encapsulated within droplets. (**b**) Histogram of the size distributions of droplets in the emulsion. (**c**) Encapsulated oligos are amplified by primers targeting the constant regions. DNA electropherogram confirms appropriately sized cDNA. (**d**) Micrographs of ΦX174 DNA co-encapsulated with digital droplet PCR reagents in brightfield and GFP channels for an emulsion without any ΦX174 DNA and where ΦX174 DNA is expected to be present in 30% of droplets. (**e**) Histogram of fluorescence intensity within droplets for the 30% positive emulsion. (**f**) Poisson estimator *λ* as a function of the percentage of positive droplets *p*, with an overlaid regression.

Encapsulating DNA in droplets is necessary for a broad array of applications, including digital PCR, enzyme screening, and single cell sequencing^1,8,9^. To demonstrate the ability of our approach to generate oligo-containing droplet libraries that can be used for downstream assays, we generate a library comprising 192 unique primer sequences (Fig. 4a). The primer library consists of universal sequences flanking an 8-oligo barcode and is stored in a 384 well plate. With conventional microfluidic techniques, generating a 192-member droplet library would take over a day of round the clock operation with a single microfluidic device running at ~ 10 min per cycle, accounting for sample loading into syringes, startup of the device, and droplet generation, and would be wasteful of syringes, tubing, and labor. With our approach, we generate this library in ~ 8 h without any microfluidics or user intervention. The resulting emulsion is monodispersed, with a coefficient of variation of 3.3% (Fig. 4b). To confirm the library contains functional primers, we break the emulsion and amplify the released barcodes, obtaining products of the expected size (Fig. 4c). This demonstrates that generation of a 192-member droplet library directly from a well plate is simple and effective and that the process does not harm the oligos.

The SciFlexArrayer functions as an automated droplet generator that, in principle, can perform any reaction compatible with well plate storage, sampling, and the emulsification mechanism. Thus, it affords an accessible means by which to conduct droplet assays without microfluidic instrumentation or expertise. To demonstrate this, we use the approach to perform digital droplet PCR (ddPCR), a ubiquitous and important application that normally requires microfluidics^21^. An important consideration in performing droplet assays is the effect of the fluidic properties of different solutions on droplet generation. When generating droplets for ddPCR using our standard parameters, we find that mean diameter increases by 23% relative to water droplets and that the coefficient of variation is 4.4%. If desired, droplets of different composition can be adjusted to a desired size by tuning generation parameters, such as pulse amplitude and width. In our ddPCR assay, we use ΦX174 virus as an example target, generating droplets at different concentrations to characterize dynamic range and accuracy. Compared to the negative control, we observe fluorescent droplets when the virus is present (Fig. 4d) obtaining a bimodal distribution corresponding to negative and positive droplets (Fig. 4e). Because we load the virus at limiting dilution, encapsulation follows Poisson statistics^22^. We use the Poisson estimator to relate the number of DNA molecules per droplet to the percentage of positive droplets (Fig. 4f)^23^. This shows that ddPCR performed with the SciFlexArrayer behaves like reactions performed with conventional microfluidics. Moreover, it illustrates the promise of this approach for conducting droplet reactions without microfluidic expertise.

We present a simple approach to generate diverse droplet libraries with full automation using a commercial liquid spotter. The droplets produced by our method are similarly uniform to those produced by microfluidic devices and, thus, can be used for common droplet assays, like ddPCR. Thus, the spotter should be useful for labs wanting to conduct droplet reactions but lacking microfluidic expertise. Moreover, its ability to precisely control the diameter of every formed droplet provides a unique opportunity for size-based labeling of droplet populations that can be used in combination with fluorescence tagging approaches^24,25^. The ability to encapsulate large arrays of samples from well plates should make the approach useful as a tool for manufacturing reagents required for applications in droplet microfluidics, including droplet libraries for combinatorial chemistry applications or particle-templated droplet libraries for screening and single cell analysis^10,23^. Our approach makes monodispersed emulsification of hundreds of reagents simple and thus overcomes the major barrier to applying droplet microfluidics to applications requiring diverse reagent libraries.

## Materials and methods

### Automated droplet library generation

A SciFlexArrayer S3 (Scienion AG) is used with either a PDC40, PDC70, or PDCX capillary nozzle. Prior to printing, the capillary is cleaned by exposing it to 1 mbar of oxygen plasma for 1 m in a plasma cleaner (Harrick Plasma). PBS from a source plate is aspirated into the capillary and printed into 1 mL of HFE-7500 (3 M) oil with 2% (w/v) PEG-PFPE amphiphilic block copolymer surfactant (RAN Biotechnologies). Droplet size as a function of acoustic wave parameters is measured at the time of printing by automated imaging processing by a camera mounted onto the SciFlexArrayer. Visualization of droplet generation is performed with a Miro R311 (Vision Research) high speed camera. Collected emulsions are pipetted onto a cell counting slide and visualized on the EVOS Cell Imaging System (Thermo Fisher) and monodispersity is calculated using ImageJ. For drop size distribution comparison, a microfluidic flow-focusing device with a cross-sectional channel dimension of 40 by 40 microns is used. Syringe pumps (New Era) are used to drive 1 mL syringes (BD) filled with HFE-7500 oil with 2% (w/v) PEG-PFPE amphiphilic block copolymer or PBS into the device at flow rates of 2000 μL/h and 500 μL/h, respectively.

### Optically encoded 64-member droplet library generation

Dextran—Cascade Blue conjugate (Thermo Fisher), Dextran—Cy5 conjugate (Thermo Fisher), and Dextran—Fluorescein conjugate (Thermo Fisher) are diluted in PBS to the following 4 concentrations: 3 μM, 15 μM, 30 μM, and 60 μM. All possible combinations of these three dyes and concentrations are mixed individually into 64 wells on a 384 well plate. A print routine is set up with a PDC70 capillary on the SciFlexArrayer to print 4000 drops of each mixture into 1 mL HFE-7500 oil with 5% (w/v) PEG-PFPE amphiphilic block copolymer in a 24-well plate. Collected emulsions are pipetted onto a cell counting slide and visualized on the EVOS Cell Imaging System using the DAPI, GFP, and Cy5 filter cubes (Thermo Fisher).

### Fluorescence image analysis

A composite image of the three channels is cropped to include an 800-pixel diameter circle. Droplet size is analyzed and those droplets that are 2 standard deviations below the mean are excluded from downstream analyses. The intensity values at the center of each droplet in each channel is recorded. The intensity histograms for each of the 3 fluorescence channels is modeled as a mixture of 4 Gaussian distributions. Droplets with intensity values that are 1.5 standard deviation above or below the mean of the nearest Gaussian are filtered. tSNE clustering is performed with the sklearn Python package.

### 192-member primer droplet library

Custom oligonucleotides are ordered from IDT and kept at − 20 °C until use. The 192-primer library consists of 96 sequences of the format CGGAGCTTTGCT AACGGTCGNNNNNNNNTCGTCGGCAGCGTCAGATGTGTATAAGAGACAG and 96 of the format CTTACGGATGTTGCACCAGCNNNNNNNNGTCTCGTGGGCTCGGAGATGTG TATAAGAGACAG, where NNNNNNNN is a randomized 8 bp barcode that is a Hamming distance of at least 3 from all other barcodes. Each oligo is diluted to 5 μM in water and printed using a PDC70 into 1 mL HFE-7500 (3 M) oil with 5% (w/v) PEG-PFPE amphiphilic block copolymer in a 24-well plate. A 2-mm diameter stir bar (V&P Scientific) is added into the well during printing. After printing, the collected emulsion is broken with an equal volume of 20% (v/v) perfluoro-1-octanol (Sigma-Aldrich) in HFE-7500. The emulsion is amplified using 1 × KAPA HiFi HotStart ReadyMix (Roche) and 1 μM of forward primer and reverse primer (IDT). The thermocycling conditions are: 95 °C for 3 m; 8 cycles of 95 °C for 20 s, 60 °C for 30 s, 72 °C for 20 s; and a final extension of 5 m at 72 °C. cDNA is purified using a 2 × sample volume ratio of AMPure XP (Beckman Coulter) beads and analyzed on the Agilent 2100 Bioanalyzer.

### Digital droplet PCR

PhiX-174 virion DNA (New England Biolabs) is mixed with PCR reagents containing 1X Platinum Multiplex PCR Master Mix (Life Technologies), 200 nM probe (IDT), 1 μM forward primer (IDT), 1 μM reverse primer (IDT), 0.5% (v/v) Tween 20 (Sigma-Aldrich), and 2.5% (w/v) Poly(ethylene glycol) 6000 (Sigma-Aldrich). The reaction mix is printed with a PDC70 capillary into 100 μL HFE 7500 oil with 5% (w/v) PEG-PFPE amphiphilic block copolymer in a 0.2 mL PCR tube. After printing, the oil is replaced with 50 μL FC-40 oil (Sigma-Aldrich) with 5% (w/v) PEG-PFPE amphiphilic block copolymer. The emulsion is amplified using the following program on a Bio-Rad T100 thermocycler: 2 m 30 s at 95 °C; 35 cycles of 30 s at 95 °C, 1 m 30 s at 60 °C, and 30 s at 72 °C; and a final extension of 5 m at 72 °C. The emulsion after thermocycling is imaged on the EVOS Cell Imaging System in brightfield and GFP channels. Intensity data is extracted from each droplet; coalesced droplets with a diameter greater than 80 μm were excluded from analysis. The Poisson estimator was calculated from the observed fraction of positive droplets by the following equation: $\lambda = -ln(1 - p)$^23^.

## Supplementary Information

Supplementary Movie S1.

Supplementary Movie S2.

Supplementary Figures.

## Data Availability

All data generated or analyzed during this study are included in this published article and supplementary information.
