# Supplementary material for: High diversity droplet microfluidic libraries generated with a commercial liquid spotter
Source: Sci Rep. 2021 Feb 23;11:4351. doi: 10.1038/s41598-021-83865-y (PMC7902812; doi:10.1038/s41598-021-83865-y)
Supplement: Supplementary file 3 — Supplementary Figures. [file 41598_2021_83865_MOESM3_ESM.docx]

**Supplementary information for:**

**High diversity droplet microfluidic libraries generated with a commercial liquid spotter**

Jesse Q. Zhang^1,2^, Christian A. Siltanen^1,3,&^, Ata Dolatmoradi^1,&^, Chen Sun^1^, Kai-Chun Chang^1^, Russell H. Cole^3^, Zev J. Gartner^2,4,5^, Adam R. Abate^1,2,5,6,*^

^1^ Department of Bioengineering and Therapeutic Sciences, University of California San Francisco, San Francisco, CA, USA

^2^ UC Berkeley-UCSF Graduate Program in Bioengineering, University of California San Francisco, San Francisco, CA, USA

^3^ Scribe Biosciences, Inc. San Francisco, CA, USA

^4^ Department of Pharmaceutical Chemistry, University of California San Francisco, San Francisco, CA, USA.

^5^ Chan Zuckerberg Biohub, San Francisco, CA, USA

^6^ California Institute for Quantitative Biosciences, University of California San Francisco, San Francisco, CA, USA

^*^ Corresponding author: [adam@abatelab.org](about:blank)

^&^ These authors contributed equally to this work.


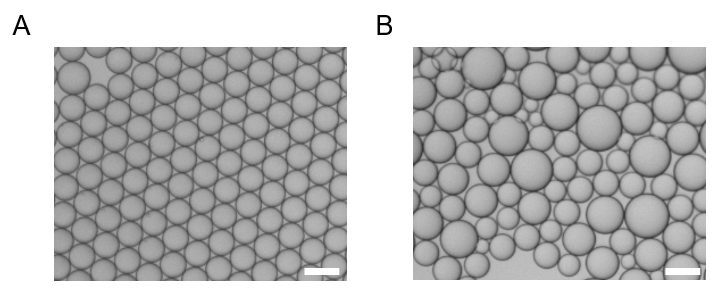


**Figure S1**. Agitating oil bath with stir bar during printing reduces coalescence. Water droplets are ejected into oil containing 2% surfactant using a PDC70 capillary with (A) or without (B) incorporation of active stirring. Scale bar = 100 um.


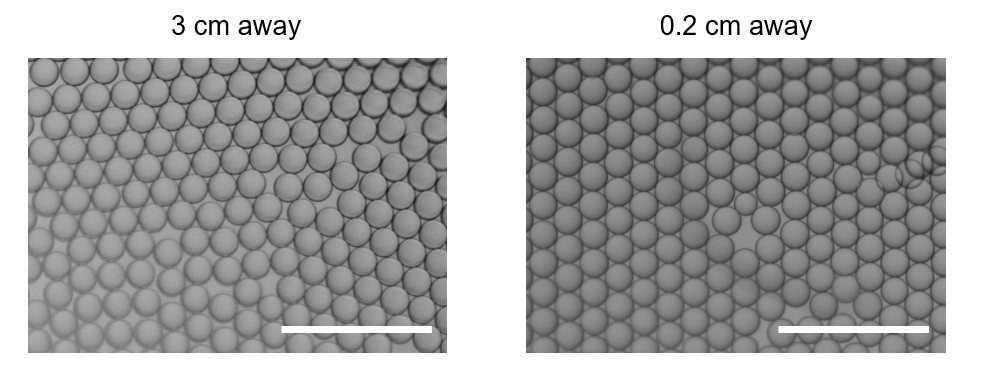


**Figure S2**. Droplet printing is robust to distance of nozzle away from oil layer. Micrographs of water droplets printed at two different nozzle heights above the oil bath. Scale bars = 400 um.


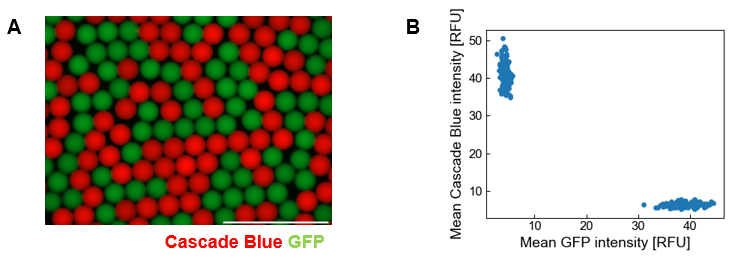


**Figure S3**. Minimal cross-over from previous samples occurs when operating the printer. We print four samples containing either 10 uM Cascade Blue (CB) or 10 uM FITC in the following order: FITC, CB, FITC, CB. Between samples, the printer performs a standard wash procedure. After imaging the resulting emulsion (A) and analyzing droplet intensities (B), we observe two clusters. Scale bar = 400 um.


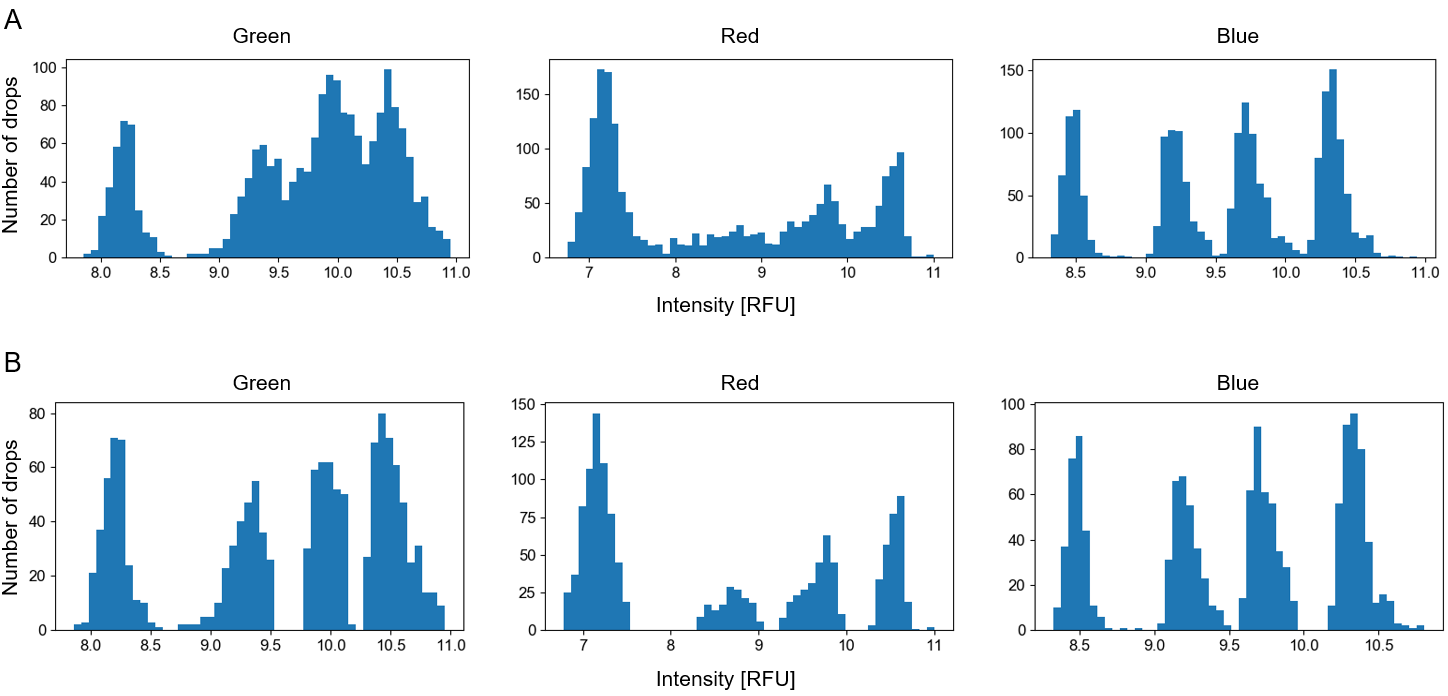


**Figure S4.** Fluorescence intensity histograms for analyzed droplets in each of the 3 imaged channels. (A) Droplet distributions without filtering. N=1958 droplets. (B) Droplet distributions after filtering out droplets in between peaks. N=1358 droplets.
